# Serotypes, seasonal trends, and antibiotic resistance of non-typhoidal *Salmonella* from human patients in Guangdong Province, China, 2009–2012

**DOI:** 10.1186/s12879-015-0784-4

**Published:** 2015-02-12

**Authors:** Zhaoming Liang, Bixia Ke, Xiaoling Deng, Junhua Liang, Lu Ran, Lingling Lu, Dongmei He, Qiong Huang, Changwen Ke, Zhongjie Li, Hongjie Yu, John D Klena, Shuyu Wu

**Affiliations:** Guangdong Provincial Institute of Biological Products and Materia Medica, Guangzhou, Guangdong China; Guangdong Provincial Centre for Disease Control and Prevention, Guangzhou, Guangdong China; Office of Disease Control and Emergency Response, Chinese Center for Disease Control and Prevention, Beijing, China; China-US Collaborative Program on Emerging and Re-emerging Infectious Diseases, U.S. Center for Disease Control and Prevention, Beijing, China; Global Disease Detection Branch, Division of Global Health Protection, Center for Global Health, U.S. Centers for Disease Control and Prevention, Atlanta, GA USA

## Abstract

**Background:**

Non-typhoidal *Salmonella* is a common cause of infectious diarrhea in humans. Antimicrobial-resistant *Salmonella* has become a global concern.

**Methods:**

Using laboratory-based surveillance system for *Salmonella* from September 2009 to December 2012 in Guangdong Province of China. The clinical information and samples of diarrhea patients were collected, according to the surveillance case definition. The lab tests were followed by standardized protocols, including sample isolation, isolates confirmation, serotyping, and antimicrobial susceptibility testing (AST).

**Results:**

A total of 1,826 *Salmonella* isolates were identified from40,572 patients in 28 hospitals in11 prefectures. The isolates ratio was highest in autumn (38.8%, 708/1826) and lowest in winter (6.4%, 117/1826). Children aged <5 years were the group most affected by *Salmonella* in Guangdong Province accounting for 73% (1,329/1,826), of whom the infants (<1 year) were 81.5% (1084/1329) especially. A total of 108 serotypes were identified among the isolates. *S*. Typhimurium represented the most common serotype followed by serotype 4,5,12:i:-.

*S.* Typhimurium was also the common serotype followed by *S.* Enteritidis among infants and children aged 1-3 years old. However, *S.* Enteritidis became the common serotype followed by *S.* Typhimurium among children aged 3–5 and >5 years.

Resistance to at least one antimicrobial was found in 72% (1321/1,826) of the isolates. Resistance to at least three antimicrobials was found in 46% (850/1,826) of the isolates. Resistance to all 12 antimicrobials screened was observed in 8 isolates (0.44%, 8/1,826). The resistant prevalence to quinolones including nalidixic acid and ciprofloxacin was 61.9% (1131/1826), of which ciprofloxacin resistance rate was 8.05% (147/1826). The prevalence resistance to all three cephalosporin antimicrobials (cefepime, cefotaxime, and caftazidime) in <5 yr age group was accounted for 90% (89/99).

**Conclusions:**

Additional data and more refined methods can improve future surveillance. The invasive Salmonella isolates should also be included to the antibiotic resistance surveillance for clinical care or public health.

## Background

Foodborne disease is a global public health problem. Non-typhoidal *Salmonella *(NTS) remains one of the most commonly reported bacterial causes of foodborne infections diseases [1]. It is well-recognized that salmonellosis can be acquired by consumption of contaminated water, meat, eggs, or milk, or by contact with infected food animals and patients [2]. Manifestations of NTS included diarrheal and septicemia. Estimates suggest that globally 93.8 million people are infected by NTS, with 155,000 deaths each year [3]. In the United States, NTSis estimated to cause 1,027,561 illnesses, 19,336 hospitalizations and 378 deaths annually [4]. It has also been shown that children aged <5 years are a susceptible population to *Salmonella* infection [5]. However, similar data with respect to NTS infections is lacking in China [6]. NTS is typically self-limiting; antimicrobials are generally reserved for invasive infections [7]. Fluoroquinolones are among the antimicrobials commonly used to treat invasive salmonellosis among adults while cephalosporins are often employed for children [8,9]. However, several studies have shown a decreased susceptibility to ciprofloxacin as well as cephalosporins in *Salmonella* [9-12]. Furthermore, fluoroquinolone treatment failure in *Salmonella* infection has been reported in some countries [13-15]. Antimicrobial -resistant *Salmonella* has become a global concern [16,17].

Currently, the national notifiable infectious diseases reporting system required diarrhea case reporting based on symptoms only. Laboratory-based surveillance is not commonly conducted at this time in China [18]. However, laboratory-based surveillance can provide information that permits identification of risk factors and control of infectious diseases, and implementation of NTS surveillance will allow for investigation of characteristics of *Salmonella* infections. Guangdong Province is a subtropical area in south China, with a season of high temperatures and humidity from March to October. Since 2009, laboratory-based surveillance for NTS has been established by the Guangdong Center for Disease Control and Prevention (GDCDC) [19]. In this report,epidemiological characteristics,including seasonal tendency, age distribution associated with serotype diversity, and antimicrobial resistance of NTS infections in Guangdong province are presented.

## Methods

### Study design

The surveillance case definition was defined as a person who presented with three or more loose stools within 24 hours and whose diarrhea was associated with one or more of the following symptoms:fever, vomiting, or abdominal pain. Data from September 2009 to December 2012 collected from 28 hospitals in 11 prefectures in Guangdong Province were included in this analysis. Among the 28 hospitals, five were pediatric facilities and the remaining were general hospitals. Epidemiologic information about the patients such as sex, age and consult date were recorded. Spring was defined from March to May, summer from June to August, autumn from September to November, winter from December to February. A standardized protocol was distributed to the sentinel hospitals to guide the collection and processing of stool samples. Clinical laboratories were responsible for *Salmonella* isolation and suspected isolates were stored in semi-solid agar and submitted to GDCDC within one week of collection for confirmation, serotyping, and *Antimicrobial susceptibility testing*.

The research involving human subjects have been approved with Guangdong medical ethics comply with the Helsinki Declaration. All the participants have approved the study.

### *Salmonella* serotyping

NTS isolates were confirmed using API 20E test strips (bioMerieux, Marcy L’Etoile, France) before serotyping. O and H antigens were characterized using commercial antiserum (S&A Reagents Lab, Bangkok, Thailand) and the serotypes were identified according to Kauffmann-White Scheme [20].

### Antimicrobial susceptibility testing (AST)

The antimicrobial susceptibility of the isolates was determined according to the guidelines of the Clinical and Laboratory Standards Institute (CLSI) [21]. Disk diffusion assays were performed on Muller-Hinton agar with antibiotic impregnated disks. The tested antibiotics included streptomycin 10 μg (S), gentamicin 10 μg (G), ceftazidime 30 μg (Caz), cefotaxime 30 μg (Ctx), cefepime 30 μg (Fep), ampicillin 10 μg (A), nalidixic acid 30 μg (Nal), ciproflaxin 5 μg (Cp), tetracycline 30 μg (T),chloramphenicol 30 μg (C), sulfamethoxazole 300 μg (Su), and trimethoprim 5 μg (Tm). The diameter of the inhibition zone was interpreted as resistant, intermediate or susceptible according to CLSI guidelines [21]. *Escherichia coli* ATCC 25922 was used as quality control organism. Ampicillin, trimethoprim, sulfamethoxazole, cefotaxime, ceftazidime, cefpime, ciprofloxacin and gentamicin were defined as clinically important antimicrobials [22]. Multi-drug resistance (MDR) was identified as resistance to three or more than three classes of antimicrobials. ACSSuT pattern was defined as resistance to ampicillin, chloramphenicol [6]. Streptomycin, sulfamethoxazole, tetracycline.

### Data analysis

Data were analyzed using SPSS version 13.0 (SPSS Institute, city, state, USA). Chi-squared analysis was performed to test the statistical association between serotypes and age distribution, and to compare the percentage of multi-drug resistant isolates in a given serotype. A *P*-value <0.05 was considered statistically significant.

## Results

### Isolation rate and serotypes

Stool samples were collected from 40,572 patients with diarrhea in Guangdong Province between September 2009 and December 2012, and1,826 NTS isolates were recovered. The isolation rate of NTS was 4.5%. A total of 108 serotypes were identified among the isolates. Ten serotypes accounted for 80% (1,462/1,826) of the NTS isolates in this study, including *S.* Typhimurium (n = 550; 30%), *S.* serotype 4,5,12:i:- (n = 254; 14%), *S.* Enteritidis (n = 245; 13%), *S.* Stanley (n = 189; 10%), *S.* Derby (n = 48; 2.6%), *S.* Rissen (n = 43; 2.4%), *S.* Weltevreden (n = 38; 2.1%), *S.* Infantis (n = 29; 1.6%), *S.* Thompson (n = 26; 1.4%), *S.* Albany (n = 20; 1.1%) and *S.* Agona (n = 20; 1.1%).

### Seasonal, sex and age distribution

The isolation rate in spring was 3.5% (261/7,488), 6.4% (680/10594) in summer, 5.4% (708/13,039) in autumn and 1.9% (177/9,451) in winter. The isolation rate was highest in September 2011 (10%, 83/817) and lowest in December, 2009 (0.8%, 7/861). Infections occurring in autumn accounted for 39% of all NTS cases followed by 37% in summer.

There were 1,031 males and 725 females infected with NTS; the sex of 70 cases was unknown. The overall male/female ratio was 1.4:1. The patients ranged in age from 20 days to 96 years (115 cases unknown). The median age was 1 year. Children <5 years accounted for73% (1,329/1,826) of NTS cases. Of the 1,329 patients,1,084 were infants <1 year. Among infants, *S.* Typhimurium was the most common serotype causing 37% (404/1,084) of infections followed by *S.* serotype 4,5,12:i:- (17%, 189/1,084) and *S.* Stanley (11%, 120/1084). *S.* Enteritidis accounted for only 6.8% (74/1,084). Among children aged1-2 and 2–3 years, *S.* Typhimurium was the highest, followed by *S.* Enteritidis, *S.* serotype 4,5,12:i:- and *S.* Stanley. However, among children aged 3–4, 4–5, and >5 years, the proportion infected with *S.* Enteritidis became the highest compared to other serotypes, and *S.* Typhimurium became the second common serotype (Table 1).

**Table 1:** **Descriptive data of** ***Salmonella*** **infections by serotype in diarrhea patients in Guangdong province in 2009 – 2012**

**Variable**	**Typhimurium**	**4,5,12:i:-**	**Enteritidis**	**Stanley**	**Derby**	**Rissen**	**Weltevreden**	**Infantis**	**Thompson**	**Albany**	**Agona**	**Other**	**Overall**
	**N (%)**	**N (%)**	**N (%)**	**N (%)**	**N (%)**	**N (%)**	**N (%)**	**N (%)**	**N (%)**	**N (%)**	**N (%)**	**N (%)**	**N (%)**
Season													
Spring	100 (38)	35 (13)	41 (16)	23 (8.8)	6 (2.3)	4 (1.5)	7 (2.7)	0 (0.0)	6 (2.3)	1 (0.4)	2 (0.8)	36 (14)	261 (100)
Summer	184 (27)	111 (16)	88 (13)	59 (8.7)	23 (3.4)	23 (3.4)	10 (1.5)	10 (1.5)	5 (0.7)	10 (1.5)	12 (1.8)	145 (21)	680 (100)
Autumn	213 (30)	87 (12)	88 (12)	96 (14)	14 (2.0)	13 (1.8)	14 (2.0)	18 (2.5)	11 (1.6)	9 (1.3)	5 (0.7)	140 (20)	708 (100)
Winter	53 (30)	21 (12)	28 (16)	11 (6.2)	5 (2.8)	3 (1.7)	7 (4.0)	1 (0.6)	4 (2.3)	0 (0.0)	1 (0.6)	43 (24)	117 (100)
Sex													
Male	324 (31)	151 (15)	125 (12)	102 (10)	27 (2.6)	24 (2.3)	25 (2.4)	16 (1.6)	15 (1.5)	8 (0.8)	12 (1.2)	202 (20)	1031 (100)
Female	200 (28)	98 (14)	109 (15)	81 (11)	20 (2.8)	16 (2.2)	12 (1.7)	11 (1.5)	8 (1.1)	12 (1.7)	8 (1.1)	150 (21)	725 (100)
Unknown	26 (37)	5 (7.1)	11 (16)	6 (8.6)	1 (1.4)	3 (4.3)	1 (1.4)	2 (2.9)	3 (4.3)	0 (0.0)	0 (0.0)	12 (17)	70 (100)
Age													
≤1	404 (37)	189 (17)	74 (6.8)	120 (11)	24 (2.2)	26 (2.4)	13 (1.2)	18 (1.7)	13 (1.2)	10 (0.9)	10 (0.9)	183 (17)	1084 (100)
~2	41 (21)	32 (17)	34 (18)	35 (18)	5 (2.6)	2 (1.0)	0 (0.0)	2 (1.0)	5 (2.6)	3 (1.6)	4 (2.1)	29 (15)	192 (100)
~3	24 (28)	10 (9.1)	24 (22)	7 (6.4)	4 (3.6)	1 (0.9)	7 (6.4)	3 (2.7)	1 (0.9)	0 (0.0)	2 (1.8)	27 (24)	110 (100)
~4)	12 (17)	3 (4.3)	22 (32)	6 (8.7)	1 (1.4)	1 (1.4)	4 (5.8)	0 (0.0)	2 (2.9)	3 (4.3)	1 (1.4)	14 (20)	69 (100)
~5	9 (19)	4 (8.5)	18 (38)	2 (4.3)	1 (2.1)	0 (0.0)	1 (2.1)	1 (2.1)	0 (0.0)	1 (2.1)	0 (0.0)	10 (21)	47 (100)
≥5	20 (9.6)	7 (3.3)	54 (26)	11 (5.3)	10 (4.8)	9 (4.3)	12 (5.7)	3 (1.4)	2 (1.0)	3 (1.4)	3 (1.4)	75 (36)	209 (100)
Unknown	40 (35)	9 (7.8)	19 (16)	8 (7.0)	3 (2.6)	4 (3.5)	1 (0.9)	2 (1.7)	3 (2.6)	0 (0.0)	0 (0.0)	26 (23)	115 (100)
Overall	550 (30)	254 (14)	245 (13)	189 (10)	48 (2.6)	43 (2.4)	38 (2.1)	29 (1.6)	26 (1.4)	20 (1.1)	20 (1.1)	364 (20)	1826 (100)

### Antimicrobial resistance

A total of 168 (9.2%) of the 1,826 NTS isolates were susceptible to all 12 antimicrobials (9.2%). *S*. Weltevreden showed a high rate of pan-susceptibility to antimicrobials (47%, 18/38), followed by *S*. Stanley (31%, 58/189). However, very few *S*. Typhimurium (1.5%; 8/550) and *S.* serotype 4,5,12:i:- (0.8%,2/254) isolates were pan-susceptible. The *Salmonella* isolates obtained in this study showed high prevalence of resistance to the tested antimicrobials (Table 2). Overall NTS isolates from children aged < 5 years showed a higher prevalence of antimicrobial resistance than those from patients >5 years, and included resistance to cephalosporins and quinolones.

**Table 2:** **Antimicrobial resistance of** ***Salmonella*** **serotypes in Guangdong province in 2009 - 2012**

**Antimicrobial**	**Typhimurium (n = 550)**		**4,5,12:i:- (n = 254)**		**Enteritidis (n = 245)**		**Stanley (n = 189)**		**Derby (n = 48)**		**Rissen (n = 43)**	
	**<5 yrs**	**≥5 yrs**	**<5 yrs**	**≥5 yrs**	**<5 yrs**	**≥5 yrs**	**<5 yrs**	**≥5 yrs**	**<5 yrs**	**≥5 yrs**	**<5 yrs**	**≥5 yrs**
	**N (%)**	**N (%)**	**N (%)**	**N (%)**	**N (%)**	**N (%)**	**N (%)**	**N (%)**	**N (%)**	**N (%)**	**N (%)**	**N (%)**
Aminoglycosides												
Getamicin	302 (66)	32 (65)	131 (57)	10 (71)	14 (12)	13 (12)	11 (6.9)	1 (4.8)	8 (27)	6 (40)	3 (11)	3 (27)
Streptomycin	335 (73)	36 (74)	154 (67)	9 (64)	44 (36)	41 (39)	22 (14)	1 (4.8)	10 (33)	7 (47)	13 (46)	6 (54)
Cephalosporins												
Cefepime	71 (15)	3 (6.1)	29 (13)	1 (7.1)	14 (12)	6 (5.7)	9 (5.6)	0 (0.0)	3 (10)	0 (0.0)	5 (18)	0 (0.0)
Cefotaxime	67 (14)	5 (10)	30 (13)	0 (0.0)	12 (10)	5 (4.8)	10 (6.3)	1 (4.8)	3 (10)	0 (0.0)	5 (18)	0 (0.0)
Caftazidime	45 (9.8)	2 (4.1)	18 (7.8)	0 (0.0)	7 (5.8)	5 (4.8)	8 (5.0)	1 (4.8)	1 (3.3)	0 (0.0)	3 (11)	0 (0.0)
Penicillins												
Ampicillin	412 (89)	35 (71)	215 (93)	11 (79)	68 (56)	59 (56)	18 (11)	3 (14)	12 (40)	6 (40)	21 (75)	8 (73)
Quinolones												
Nalidixic acid	383 (83)	37 (76)	167 (73)	13 (93)	94 (78)	89 (85)	17 (11)	1 (4.8)	11 (37)	7 (47)	3 (11)	1 (9.1)
Ciprofloxacin	56 (12)	5 (10)	27 (12)	1 (7.1)	7 (5.8)	7 (6.7)	8 (5.0)	0 (0.0)	1 (3.3)	3 (20)	1 (3.6)	0 (0.0)
Tetracyclines												
Tetracycline	402 (87)	34 (69)	214 (93)	13 (93)	32 (26)	28 (27)	22 (14)	1 (4.8)	21 (70)	8 (53)	25 (89)	8 (73)
Amphenicols												
Chloramphenicol	335 (73)	28 (57)	154 (67)	9 (64)	5 (4.1)	5 (4.8)	8 (5.0)	2 (9.5)	9 (30)	6 (40)	4 (14)	1 (9.1)
Sulfonamides												
Sulfamethoxazole	398 (86)	35 (71)	206 (89)	12 (86)	60 (50)	53 (50)	31 (19)	3 (14)	15 (50)	7 (47)	21 (75)	5 (46)
Trimethoprim	298 (65)	27 (55)	117 (51)	8 (57)	16 (13)	10 (9.5)	10 (6.3)	2 (9.5)	8 (27)	6 (40)	24 (86)	9 (82)
Multi-resistance												
≥3 antimicrobials	356 (77)	31 (63)	188 (81)	9 (64)	65 (54)	73 (70)	49 (31)	7 (33)	15 (50)	6 (40)	22 (79)	8 (73)
ACSSuT*	244 (53)	25 (51)	92 (40)	6 (43)	3 (2.5)	3 (2.9)	2 (1.3)	1 (4.8)	6 (20)	4 (27)	3 (11)	1 (9.1)
	**Weltevreden (n = 38)**	**Infantis (n = 29)**	**Thompson (n = 26)**	**Albany (n = 20)**	**Agona (n = 20)**	**Other (n = 364)**	**Weltevreden (n = 38)**	**Infantis (n = 29)**	**Thompson (n = 26)**	**Albany (n = 20)**	**Agona (n = 20)**	**Other (n = 364)**
	**<5 yrs**	**≥5 yrs**	**<5 yrs**	**≥5 yrs**	**<5 yrs**	**≥5 yrs**	**<5 yrs**	**≥5 yrs**	**<5 yrs**	**≥5 yrs**	**<5 yrs**	**≥5 yrs**
	**N (%)**	**N (%)**	**N (%)**	**N (%)**	**N (%)**	**N (%)**	**N (%)**	**N (%)**	**N (%)**	**N (%)**	**N (%)**	**N (%)**
Aminoglycosides												
Getamicin	3 (23)	1 (4.2)	2 (10)	2 (29)	2 (10)	1 (25)	3 (27)	1 (11)	4 (29)	1 (17)	37 (17)	19 (16)
Streptomycin	3 (23)	3 (12)	2 (10)	2 (29)	12 (63)	3 (75)	3 (27)	3 (33)	4 (29)	2 (33)	61 (28)	37 (32)
Cephalosporins												
Cefepime	3 (23)	1 (4.2)	2 (10)	2 (29)	1 (5.3)	1 (25)	4 (36)	1 (11)	3 (21)	1 (17)	20 (9.0)	19 (16)
Cefotaxime	2 (15)	0 (0.0)	3 (15)	0 (0.0)	5 (26)	1 (25)	4 (36)	0 (0.0)	2 (14)	0 (0.0)	0 (0.0)	9 (7.7)
Caftazidime	2 (15)	0 (0.0)	2 (10)	0 (0.0)	6 (32)	1 (25)	3 (27)	0 (0.0)	2 (14)	1 (17)	16 (7.2)	7 (6.0)
Penicillins												
Ampicillin	1 (7.7)	2 (8.3)	16 (80)	6 (86)	12 (63)	1 (25)	11 (100)	9 (100)	3 (21)	2 (33)	79 (36)	33 (28)
Quinolones												
Nalidixic acid	2 (15)	2 (8.3)	16 (80)	5 (71)	5 (26)	2 (50)	9 (82)	9 (100)	4 (29)	0 (0.0)	71 (32)	36 (31)
Ciprofloxacin	0 (0.0)	1 (4.2)	0 (0.0)	0 (0.0)	0 (0.0)	2 (50)	1 (9.1)	0 (0.0)	3 (21)	1 (17)	19 (8.6)	4 (3.4)
Tetracyclines												
Tetracycline	2 (15)	4 (17)	11 (55)	6 (86)	13 (68)	2 (50)	9 (82)	9 (100)	6 (43)	2 (33)	82 (37)	52 (44)
Amphenicols												
Chloramphenicol	0 (0.0)	2 (8.3)	12 (60)	6 (86)	7 (37)	2 (50)	9 (82)	9 (100)	3 (21)	2 (33)	45 (20)	16 (14)
Sulfonamides												
Sulfamethoxazole	3 (23)	5 (21)	15 (75)	6 (86)	14 (74)	2 (50)	10 (91)	9 (100)	3 (21)	1 (17)	79 (36)	50 (43)
Trimethoprim	1 (7.7)	3 (12)	13 (65)	4 (57)	8 (42)	2 (50)	9 (82)	9 (100)	2 (14)	1 (17)	51 (23)	37 (32)
Multi-resistance												
≥3 antimicrobials	4 (31)	12 (50)	13 (65)	3 (43)	16 (84)	4 (100)	9 (82)	8 (89)	5 (36)	4 (67)	106 (48)	62 (53)
ACSSuT*	0 (0.0)	0 (0.0)	1 (5.0)	2 (29)	6 (32)	1 (25)	3 (27)	3 (33)	1 (7.1)	1 (17)	25 (11)	12 (10)

*Resistance to ampicillin, chloramphenicol, streptomycin, sulfamethoxazole, tetracycline.

Resistance to cefepime, cefotaxime, and ceftazidime was found in 10%, 11%, and 7.4% of isolates, respectively. Resistance to all three cephalosporin antimicrobials was seen in 99 isolates, of which patients <5 years accounted for 90% (89/99) and *S*. Typhimurium accounted for 46% (45/99). Among children <5 years, isolates resistant to cefepime, cefotaxime and caftazidime accounted for 12%, 12%and 8.5% while the resistance rate in patients ≥5 years was 4.7%, 5.5%, and 4.5%, respectively. The resistance rate to cefepime (*P* < 0.01) and cefotaxime (*P* < 0.01) was higher in children <5 years than in patients >5 years but no difference was seen in the resistance rate to ceftazidime (*P* > 0.05).

**Figure 1:**
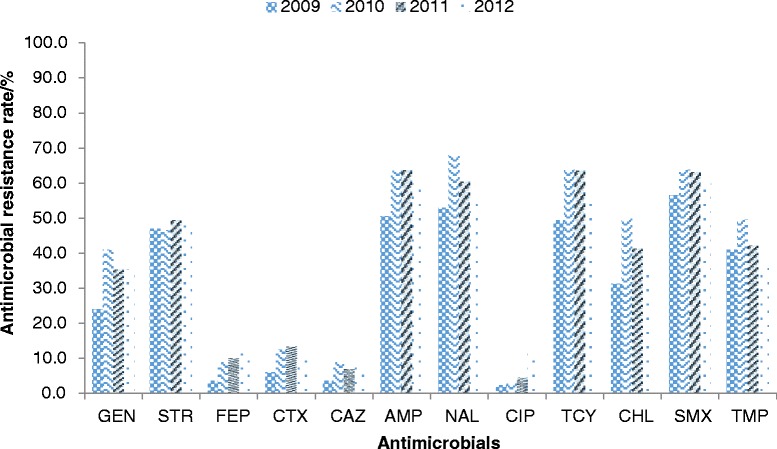
**Antimicrobial resistance rate of different antimicrobial agents inNon-typhoidal Salmonella in Guangdong, 2009–2012.**

A high prevalence of nalidixic acid resistance was observed (58%; 1,062/1,826) in the NTS isolates. A total of 155 isolates (8.5%, 155/1826) were resistant to ciprofloxacin. The overall resistance rate to ciprofloxacin increased from 2.4% in 2009 to 12% in 2012 (Figure 1). *S*. Typhimurium, *S.* serotype 4,5,12:i:- and *S*. Enteritidis accounted for 843 out of the 1,062 resistant isolates (79%). Isolates of *S*. Typhimurium made up 41% (64/155) of the ciprofloxacin-resistant isolates, followed by *S.* serotype4,5,12:i:- (19%, 30/155).

A rate of 63% (1156/1826) MDR was observed in NTS isolates in this study, with *S*. Typhimurium isolates accounting for 36% (418/1156) followed by *S.* serotype 4,5,12:i:- (18%, 206/1156) and *S*. Enteritidis (13%, 150/1156). Resistance to at least one antimicrobial was found in 72% (1321/1,826) of the isolates; resistance to at least three antimicrobials was found in 46% (850/1,826) of the isolates. Resistance to all 12 antimicrobials screened as part of this surveillance project was observed in 8 isolates (6 *S.* Typhimurium, a single *S.* serotype 4,5,12:i:- and a single Duisburg). In this study, 473NTS isolates showed the ACSSuT resistance pattern. *S*. Typhimurium accounted for 60% (284/473) followed by *S.* serotype 4,5,12:i:- (21%, 101/473). This resistance pattern was significantly associated with infection by *S.* Typhimurium and *S.* serotype 4,5,12:i:-compared to other serotypes (*P*< 0.01). Childrenaged < 5 years accounted for 27% (412/1,502) of NTS isolates with the ACSSuT pattern, while there were only 16% (33/209) patients aged ≥5 yearshad this pattern (*P*< 0.01).

## Discussion

In this study, we report epidemiologic characteristics of NTS infections in Guangdong Province from 2009 to 2012. An increase in the NTS isolation rate was observed beginning at 3.4% in 2009, 3.5% in 2010, 4.4% in 2011 and 5.1% in 2012. Since the surveillance was conducted, several training courses were designed by GDCDC for clinicians and lab staff to improve the capacity of detecting and reporting food-borne disease cases in Guangdong Province [23].

Our study showed that children aged <5 years were the group most affected by NTS in Guangdong Province. In a previous study, children <5 years were shown to be at risk for NTS infections [24]. In the United States, the NTS isolation rate was highest in patients aged < 1 year [25]. In our study, children aged <5 years accounted for 73% of the overall NTS infections. Even in general hospitals, children <5 years accounted for 65% of the infections. This rateis were higher than that in a previous national report, which NTS isolated from eight provinces were associated with diarrhea in 34% of patients < 5 years in China [6]. In other countries, a high prevalence of NTS infection in children < 5 years has also been reported [26,27]. Future epidemiological investigations about risk factors should be conducted to determine why children <5 years, especially infants (<1 year) in Guangdong have become the majority of infections for disease control and prevention.

*Salmonella* serotype 4,5,12:i:- is considered a monophasic variant of serotype Typhimurium with similar antigenic and genotypic characteristics. In our study, the percentage of *S.* serotype 4,5,12:i:- increased from 3.6% in 2009 to 20% in 2011 and down to 15% in 2012, becoming one of the most common *Salmonella* serotypes in Guangdong. In Europe,*S.* serotype 4,5,12:i:-showed a marked increase in foodborne infections with an association in pig meat [28]. This serotype has also been recognized as an emerging cause of infection in other countries in the world [29].

Increasing antimicrobial resistance was observed in this study, especially to the conventional first-line agents, such as β-lactam antibiotics and quinolones. Regionally, NTS isolates recovered in Malaysia also showed high resistance rates to tetracycline (70%), sulfonamides (57%), and streptomycin (53%) but lower rates to ampicillin (30%),nalidicix acid (28%) and chloramphenicol (21%) than in China [30]. Resistance rates in the United States to ampicillin, chloramphenicol, and nalidixicacid were even lower, 20%, 11%, and 2.7%, respectively [31]. Drug resistance in China is likely multifaceted, with resistant isolates selected after environmental, clinical and veterinary antibiotic exposures. Further investigations exploring the emergence of resistant NTS isolates are required to control this public health problem.

Extended-spectrum cephalosporins and fluoroquinolones have been recommended for treatment of diarrhea resulting from a NTS infection [32,33], although a recent review suggests that there is little added benefit for treatment in an otherwise healthy individual [33]. It is noted that the prevalence of cephalosporins resistance was higher in children aged <5 years than in patients aged ≥5 years. Also the majority of isolates resistant to all three cephalosporins were from children aged <5 years, suggesting more attention should be given to the severe resistance of *Salmonella* infections in young children. High resistance will increase the diarrhea burden among children [34]. In the United States, 4.1% of the isolates displayed decreased susceptibility to either ceftriaxone or ceftiofur in 2005 and 2006, while it was only 1% in 1996-1998 [9]. An increase in the number of isolates displaying decreased susceptibility may have an impact on clinical treatment and may lead to treatment failure as cephalosporins are the primary choice for children.

Reduced susceptibility to ciprofloxacin is a current trend in NTS isolates globally. The MIC of NTS isolates from Asian countries rose from 0.125 to 1 μg/ml during 2003 to 2005 [12]. Nalidixic acid susceptibility testing has been recommended by CLSI before using ciprofloxacin for treatment, as isolates resistant to nalidixic acid are probably resistant to ciprofloxacin [35].

This study had some limitations. First, selection of the sentinel hospitals was based on convenience but not the catchment size of the local population. Ten of the 28 hospitals involved were located in the capital city, Guangzhou, while only two or three hospitals included from the other cities in Guangdong. Future expansion of NTS surveillance in Guangdong should incorporate hospitals based on the coverage of the population in each catchment area. Second, only a minority of all patients with diarrhea have stool specimens collected for culture, as stool culture is not required in China for diarrheal patients. Finally, the *Salmonella* isolation rate was not very high, possibly because of previous antibiotic use or laboratory testing sensitivity. In the future, increasing the rate of specimen collection, the rate of isolation, the number of facilities in surveillance, and improving the representativeness of the surveillance system will help to improve our understanding of foodborne disease incidence and increase sensitivity for outbreak detection in Guangdong China.

## Conclusion

In conclusion, *S*. Typhimurium represented the most common serotype followed by serotype 4,5,12:i:- in Guangdong Province in China. Children aged <5 years were the group most affected by *Salmonella* in Guangdong Province. A high prevalence of antimicrobial resistance, including resistance to ciprofloxacin and cephalosporins, was observed among the isolates, especially from children aged <5 years. Improved characterization of *Salmonella* infections would facilitate risk assessment of *Salmonella* infections. Moreover, making the antimicrobial susceptibility testing of *Salmonella* isolates by clinical laboratories routinely would allow clinicians to choose appropriate antimicrobials for treatment of infectious diarrhea. However, additional data and more refined methods can improve future surveillance. The invasive Salmonella isolates should also be included to the antibiotic resistance surveillance for clinical care or public health.

### Limitation

It was a limitation that this study only included isolates from stool but not from blood and etc. Due to the statistic we have collected, we only can provide clinical correlates with these isolates about whether the cases are outpatients or inpatients. Since Fluoroquinolones are commonly used to treat serious diarrheal for adults while cephalosporins are often employed for children in China, we compared the resistance of cefepime, nalidixic acid, ciprofloxacin in inpatient cases is more severe than in outpatient cases (P < 0.01), respectively. If we assumed inpatient cases are more severe than outpatient cases, it seems like the high antibiotic resistance prevalence is associated with serious clinical diseases. However, more information should be collected and analyzed in the future.

## Consent

Written informed consent was obtained from the patient’s guardian/parent/next of kin for the publication of this report and any accompanying images.

## Abbreviations

NTS: Non-typhoidalSalmonella
GDCDC: Guangdong center for disease control and prevention
AST: Antimicrobial susceptibility testing
CLSI: Clinical and laboratory standards institute
MDR: Multi-drug resistance
ACSSuT: Resistance to ampicillin, chloramphenicol, streptomycin, sulfamethoxazole, tetracycline
